# Advances in measuring pediatric overall health: the PROMIS® Pediatric Global Health scale (PGH-7)

**DOI:** 10.1007/s00431-022-04408-9

**Published:** 2022-02-15

**Authors:** Michiel A. J. Luijten, Lotte Haverman, Raphaële R. L. van Litsenburg, Leo D. Roorda, Martha A. Grootenhuis, Caroline B. Terwee

**Affiliations:** 1 Emma Children’s Hospital, Amsterdam UMC, University of Amsterdam, Child and Adolescent Psychiatry & Psychosocial Care, Amsterdam Reproduction and Development, Amsterdam Public Health, Meibergdreef 9, Postbus 226601100 AD Amsterdam, the Netherlands; 2 Epidemiology and Data Science, Amsterdam UMC, Vrije Universiteit, Amsterdam, the Netherlands; 3 Princess Máxima Center for Pediatric Oncology, Utrecht, the Netherlands; 4 Pediatric Oncology, Emma’s Children’s Hospital, Amsterdam UMC, Vrije Universiteit Amsterdam, Cancer Center Amsterdam, Amsterdam, the Netherlands; 5 Amsterdam Rehabilitation Research Center | Reade, Amsterdam, the Netherlands

**Keywords:** Efficiency, Patient-reported outcomes, Outcome measurement, Psychometrics, Reliability, Validity

## Abstract

In this cross-sectional study, we aimed to assess the reliability, validity, and efficiency of the Patient-Reported Outcomes Measurement Information System (PROMIS) Pediatric Global Health scale (PGH-7) to reduce patient burden when assessing overall health in clinical practice. In total, 1082 children (8–18), representative of the Dutch population, completed the PGH-7 and the Pediatric Quality of Life Inventory (PedsQL™ 4.0), a common legacy instrument used in clinical practice to assess overall health. The assumptions for fitting an item response theory model were assessed: unidimensionality, local independence, and monotonicity. Subsequently, a model was fitted to the data to assess item fit and cultural differential item functioning (DIF) between Dutch and US children. A strong correlation (> .70) was expected between the PGH-7 and PedsQL, as both instruments measure physical, mental, and social domains of health. Percentages of participants reliably measured (> 0.90) were assessed using the standard error of measurement (SE(*θ*) < 0.32). Efficiency was calculated ((1 − SE(*θ*)^2^)/*n*_items_) to compare how well both measures performed relative to number of items administered. The PGH-7 met all assumptions and displayed good structural and convergent (*r* = .69) validity. One item displayed cultural DIF. Both questionnaires measured reliably (%*n*_PGH-7_ = 73.8%, %*n*_PedsQL_ = 76.6%) at the mean and 2SD in clinically relevant direction. PGH-7 items were 2.6 times more efficient in measuring overall health than the PedsQL.

*   Conclusion*: The PGH-7 displays sufficient validity and reliability in the general Dutch pediatric population and measures more efficiently than the PedsQL, the most commonly used legacy instrument. The PGH-7 can be used in research and clinical practice to reduce patient burden when assessing overall health.

**Table:** 

**What is Known:** *• Generic instruments which validly and reliably assess overall pediatric health are scarce.* *• Brief instruments are required for implementation of self-report patient-reported outcomes in clinical practice.*	
**What is New:** *• The PROMIS Pediatric Global Health (PGH-7) can be used in research and clinical practice to briefly assess overall pediatric health, while providing valid and reliable measurements.* *• The PGH-7 provides more efficient assessment of pediatric overall health than the Pediatric Quality of Life Inventory.*

**What is Known:**

*• Generic instruments which validly and reliably assess overall pediatric health are scarce.*

*• Brief instruments are required for implementation of self-report patient-reported outcomes in clinical practice.*

**What is New:**

*• The PROMIS Pediatric Global Health (PGH-7) can be used in research and clinical practice to briefly assess overall pediatric health, while providing valid and reliable measurements.*

*• The PGH-7 provides more efficient assessment of pediatric overall health than the Pediatric Quality of Life Inventory.*

## Introduction

The increasing interest in value-based health care and shared decision-making in pediatrics has stimulated the use of patient-reported outcome measures (PROMs) in clinical practice and research in order to monitor and screen individual patients or patient populations and to improve the quality of health care [1–3]. PROMs are questionnaires on which patients report their experiences regarding their own symptoms, functioning, quality of life, and health.

PROMs can be divided into disease-specific or generic instruments. Disease-specific instruments contain questions which are only applicable to specific conditions (such as specific symptoms). Generic (or universal) instruments are applicable to all patient populations and thus facilitate comparisons between different disease populations. In addition, generic instruments can measure either overall (or global) health or a specific domain of health (such as physical functioning). Generic PROMs that assess overall health are intended to provide a quick overview of the overall health (status) of a patient, while taking into account multiple domains [4–6]. These generic measures of overall health can be used in addition to or as a replacement of domain-specific measures to standardize the measurement of overall health across populations. Using generic instruments to measure overall health and expressing it as a single uniform score instead of assessing health with multiple domain-specific instruments allow for easier interpretation and comparisons across disease groups.

In pediatrics, a widely used generic measure of overall health(-related quality of life) is the Pediatric Quality of Life Inventory (PedsQL™ 4.0) [7–9]. The PedsQL was developed using the classical test theory (CTT) model, whereas more recently developed PROMs are developed using item response theory (IRT) modeling procedures. IRT allows items (or questions) to have a different weight in calculating the total score, which results in fewer items required to provide reliable scores than with CTT. The Patient-Reported Outcomes Measurement Information System (PROMIS®) investigators developed the generic PROMIS Pediatric Global Health Scale V1.0 (PGH-7) as a measure of global overall health using IRT modeling. The PGH-7 consists of seven items operationalizing overall health, quality of life, and physical, mental, and social health [10]. The PGH-7 has thus far shown to be a valid and reliable measure in US pediatric populations [11, 12]. The PGH-7 was selected by the International Consortium of Health Outcomes Measures (ICHOM) as part of the standard set for measuring pediatric overall health due to its short administration time and strong psychometric properties.

In this study, we assess the validity and reliability of the PGH-7 in a representative sample of the Dutch general population and assess differential item functioning (DIF) between Dutch and US children. Additionally, we compare the reliability and relative efficiency of the PGH-7 with the PedsQL in order to determine which instrument provides most information per item about the participants’ level of overall health. Finally, we provide normative data on the PGH-7 scale score.

## Materials and methods

### Procedure and participants

Children aged 8–18 years (*n* = 2654) were asked to complete the PGH-7 (*n*_items_ = 7) and the PedsQL (*n*_items_ = 23) between December 2017 and April 2018 through the marketing agency Kantar Public [13] using a two-step random stratified sampling method to prevent selection and response bias. The goal of the recruitment was to obtain a sample representative (within 2.5% on key demographics) of the Dutch general population (Gold Standard 2017—Statistics Netherlands; www.cbs.nl/en-gb). For more details on the data collection procedure, please see Luijten et al. [13]. Eighteen-year-olds did not complete the PedsQL, as normative data for 18-year-olds had previously been collected with the adult version of the PedsQL. E-mails were sent to participants by Kantar Public with a link to the study website (onderzoek.hetklikt.nu/promis), where they could log in and complete the PROMs. Due to online administration, the results were logged once all measures were completed; therefore, missing data was not permitted. Informed consent was provided by parents (children aged 8–15) and adolescents (aged ≥ 12 years). This study was approved by the Medical Ethics Testing Committee (METC) of the Amsterdam UMC.

### Measures

#### Sociodemographic questionnaire

Parents completed a sociodemographic questionnaire about themselves (age, ethnicity, and educational level) and their child (age, gender, level of education, and the presence of any chronic health conditions). For parents, the educational level was divided into low (primary, lower vocational, lower and middle general education), middle (middle vocational, higher secondary, and pre-university education), and high (higher vocational education, university).

#### PROMIS pediatric global health scale v1.0

The PROMIS Pediatric Global Health 7 scale v1.0 (PGH-7) is a generic measure that assesses physical, mental, and social health in children aged 8–18 years [10]. The scale consists of 7 items that are summarized into a single score of overall health. Participants respond to items without a recall period on how they would rate their overall health, quality of life, and their physical, mental, and social health, using a 5-point Likert scale with varying response categories. There are two additional screener items (PGH-7 + 2), which are single items from the pain interference and fatigue item banks, which do not contribute to the scale score. PGH-7 scores were calculated by applying the US IRT model to the responses of participants on the seven items, resulting in a *T* score where 50 is the mean of the US general population with a standard deviation of 10. A higher *T* score represents a better overall health.

#### Pediatric quality of life inventory (v4.0)

The Pediatric Quality of Life Inventory (PedsQL™ 4.0) is a generic questionnaire that assesses the self-reported health-related quality of life (HRQOL) of children (aged 8–18 years) [7]. It contains 23 items referring to four domains of HRQOL: physical functioning (8 items), emotional functioning (5 items), social functioning (5 items), and school functioning (5 items). The PedsQL utilizes a recall period of 1 week and the items (e.g., “Other kids/teens do not want to be my friend”) are scored from 1 (“Never a problem”) to 5 (”Almost always a problem”). The response options are transformed into values of 0, 25, 50, 75, and 100, respectively. Domain scores are calculated as the mean of all items in a specific domain (range 0–100, higher score represents better functioning). The PedsQL total score is calculated by the mean of all items of the entire questionnaire (range 0–100). The PedsQL has been validated for use in clinical practice in the Netherlands [14, 15].

### Statistical analyses

To assess the psychometric properties of the PGH-7, the structural and construct validities were assessed, which reflects if the PGH-7 measures what it intends to measure. Subsequently, we assessed the reliability of measurements when administering the PGH-7 and compared this to the PedsQL, to determine which instrument is more efficient in measuring overall health.

#### Structural validity

Structural validity was assessed by applying a graded response model (GRM) to the response patterns of all participants. GRM is a specific case of IRT models for ordinal data and three assumptions of the response data have to be met before a GRM model can be fitted: unidimensionality, local independence, and monotonicity [16–20] (see Table 3 in Appendix B for statistical tests and criteria).

A GRM was fitted to the data to estimate discrimination and threshold parameters with the expectation–maximization (EM) algorithm within R using “mirt (v1.29)” [21]. The *discrimination parameter* (*α*) of each item reflects the ability of an item to distinguish participants, with different levels of global health, from each other. The better each response category of each item in a questionnaire discriminates participants from each other (higher *α*), the more information the item provides for each individual participant and the less items are required for a reliable measurement. Each item thus provides a certain amount of information and the cumulative sum of all item information is known as the total test information. The four *threshold parameters* of an item with 5 response options represent the level of functioning required to change the response from one response category to the next, higher response category. Item fit was assessed by calculating the differences between observed and expected responses (given the beforementioned item parameters) under a GRM, using the *S*-*X*^2^ statistic [22]. A *p* value of the *S*-*X*^2^ statistic > 0.001 indicates that the item fits well [23]. When item misfit was present, item fit plots were examined.

#### Reliability

Reliability and relative efficiency were calculated using IRT models calibrated to both the PGH-7 and PedsQL. When applying IRT modeling, the reliability of a questionnaire is measured per individual response pattern. Each response pattern results in a different level of functioning (theta, *θ*) with an associated reliability (based on total test information) which is expressed as the standard error of the theta (SE(*θ*)). A SE(*θ*) < 0.32 corresponds to a reliability of 0.90 or higher and was considered reliable enough for the purpose of monitoring individual patients. The *θ* and SE(*θ*) of each response pattern were calculated using the Expected A Posteriori (EAP) estimator in “mirt (v1.29)” [21]. Since SE(*θ*) is based on a single measurement, it should be considered a parameter of internal consistency (not test–retest reliability), comparable to Cronbach’s alpha under CTT. As the PedsQL was developed under CTT, Cronbach’s alphas were calculated for the PedsQL and the PGH-7.

#### Relative efficiency

To provide a comparison of both instruments under the same measurement model, the *θ* estimates and SE(*θ*) were calculated for both questionnaires and presented in a reliability plot. In addition, the number of reliably estimated participants (SE(*θ*) < 0.32) between the two questionnaires was compared and the relative efficiency between the two questionnaires was calculated. The mean efficiency of a questionnaire was calculated by dividing the total test information by the number of items that were administered for each participant, and averaging the results [24]. The relative efficiency was then calculated by dividing the mean efficiency of the PGH-7 by the mean efficiency of the PedsQL.

#### Construct validity

To assess construct validity, the *T* score of the PGH-7 was correlated with the PedsQL total score. A strong correlation (*r* > 0.70) was expected as both total scores represent a measure of self-reported overall health. Lower correlations (∆*r* > 0.10) were expected with subscales of the PedsQL, as the PGH-7 intends to measure overall health.

#### Differential item functioning

To ensure that the item parameters used to calculate *T* scores were applicable to all participants, differential item functioning was assessed for age groups (8–12 and 13–18), between genders, and between the Dutch sample and US calibration sample (*N* = 3635) by a logistic ordinal regression model (“lordif” (v0.3–3)). Uniform and non-uniform DIF were evaluated using McFadden’s pseudo-*R*^2^, where a *R*^2^ ≥ 0.02 indicated DIF.

#### Normative data and cutoff values

Next, *θ* estimates and SE(*θ*) were calculated for the PGH-7 using the US model parameters (using an online HealthMeasures Scoring Service program, provided by the US Assessment Center) to calculate *T* scores. This was done to provide Dutch normative data on the US metric, as in practice the PGH-7 will be administered and scored using the US model parameters, in accordance with PROMIS conventions. Normality of the *T* score distribution was assessed using QQ-plots; when the scores were normally distributed, means were reported; otherwise, the median was reported. Cutoffs for the PGH-7 were determined by examining the *T* scores of percentiles for good (≥ 26th percentile), fair (6th–25th percentiles), and poor (≤ 5th percentile) functioning.

## Results

In total, a representative sample (within 2.5% of the Dutch general population on key demographics [13]; see Table 2 in Appendix A) of 1082 children completed the PGH-7 and the PedsQL (response rate = 1082/2654 = 40.8%), of which 98 were 18-year-olds who did not complete the PedsQL. Their sociodemographic characteristics are presented in Table 1.

**Table 1:** Sociodemographic characteristics of both PGH-7 analysis samples

	**Global health analysis sample (*n*** **= 1082)**	**Relative efficiency analysis sample (*n*** **= 984)**
	***M (SD)***	***M (SD)***
**Age (years)**	13.6(3.1)	13.2(2.8)
***T*** **score**	48.3(9.8)	48.4(9.7)
**Gender**	%	%
Male	51.4	51.1
Female	48.6	48.9
**Ethnicity**	%	%
Dutch	81.5	82.0
Western immigrant	15.9	13.7
Non-western immigrant	2.6	4.3
Educational level (parents)*	%	%
Low	12.8	12.5
Medium	48.2	48.0
High	38.9	39.5

^*^Low: primary, lower vocational, lower, middle general education; **middle**: middle vocational, higher secondary, pre-university education; **high**: higher vocational education, university

### Structural validity

The response data met the assumptions for fitting a GRM (see Table 3 in Appendix B). Thresholds ranged from − 8.25 to 0.80 and discrimination parameters ranged from 0.80 to 4.24. Three items displayed item misfit (*p* value < 0.0001): “In general, would you say your quality of life is: (Global02R1),” “In general, how would you rate your physical health? (Global03R1),” and “How often do you feel really sad? (PedGlobal2R1).” Misfit in this case indicates that some participants with lower *T* scores selected higher response categories (or the other way around) on this item than the GRM expects based on the probabilities from the entire sample.

### Reliability

**Fig. 1:**
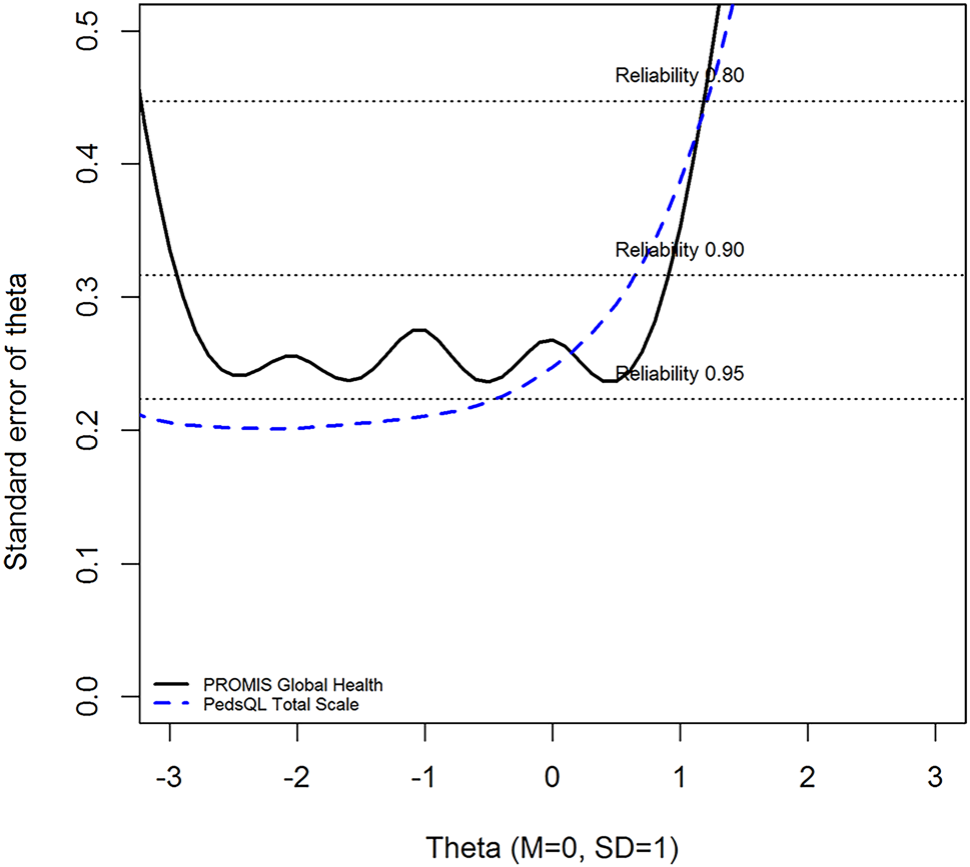
Reliability (expressed as standard error of theta) of the PROMIS Pediatric Global Health scale v1.0 and the Pediatric Quality of Life Inventory (v4.0) total score across the range of theta

Both the Dutch and US IRT models provided reliable measurements at the mean of the sample (*θ* = 0, *θ* =  − 0.17) and more than two standard deviations (SD) in the clinically relevant direction from the mean, indicating sufficient reliability (see Fig. 1). Cronbach’s alpha values were 0.87 and 0.92 for the PGH-7 and PedsQL, respectively.

### Relative efficiency

The reliability plot of the SE(*θ*) of PedsQL and PGH-7 is presented in Fig. 1.

The PedsQL outperformed the PGH-7 in terms of reliability of measurements across theta and the number of reliably estimated participants (76.6% vs. 73.8%). The relative efficiency of the PGH-7 as compared to the PedsQL was 2.6. This indicates that the PGH-7, on average, offers 2.6 times more information (as in test information) per item about the participants than the PedsQL.

### Construct validity

The PGH-7 scale *T* scores correlated moderately high (*r* = 0.69) with the PedsQL total score. The PGH-7 correlated lower (∆*r* > 0.10) with the physical (*r* = 0.50), emotional (*r* = 0.54), social (*r* = 0.49), and school (*r* = 0.49) functioning subscales of the PedsQL.

### Differential item functioning

No DIF was found for age group or gender. The item “How often do your parents listen to your ideas?” displayed uniform cross-cultural DIF (*R*^2^ = 0.027). Dutch children more often endorsed higher item response categories on this item.

### Normative data and cutoff values

*T* scores were normally distributed. The average *T* score of the PGH-7 in the Dutch population was 48.3 (SD 9.8) with a range of 19.7 to 65.8, where 5.5% of the participants received the highest possible score (65.8). Severity cutoffs were determined, where a *T* score > 40.3 indicates good global health, 33.7–40.2 indicates fair global health, and ≤ 33.6 is indicative of poor global health.

## Discussion

The PGH-7 measures validly and reliably at the mean of the Dutch population and at least two SD in the clinically relevant direction (i.e., worse global health). The reliability of the PGH-7 was comparable to the PedsQL with fewer items administered, indicating a higher efficiency. The Dutch general population reported slightly lower levels of global health (48.3) than the US population (50).

The developmental study of the PGH-7 [10] reported similar model fit, item parameters, and reliability (internal consistency) as the current study. No DIF was found between the US and Dutch model parameters except for the item “How often do your parents listen to your ideas?” This item has a skewed effect in its threshold parameters (with the lowest threshold being − 8.25) as only two participants selected the lowest item response for this item. This could be a possible explanation for the cross-cultural DIF. Therefore, we consider the US parameters to be applicable to the Dutch population.

The PedsQL is currently one of the most applied PROMs in the world for measuring overall HRQoL in pediatrics [8, 9]. One of the intended goals of the original development of the PGH-7 was to assess a child’s overall evaluation of their health (across physical, mental, and social health) faster and more efficiently than the PedsQL [10]. In this study, we were able to make a direct comparison between the two instruments, by collecting data on both instruments in the same participants. For this study, we purposely calculated reliability (internal consistency) coefficients stemming from both IRT and CTT. Both instruments performed sufficiently regarding the internal consistency and the PedsQL outperformed the PGH-7 by providing more reliable measurements, although the difference was small (76.6% vs. 73.8%). However, when looking at reliability, it is important to take the number of items administered into account. It is well-known that internal consistency increases and standard errors decrease, as more items are administered. However, the amount of items in a questionnaire are preferably limited for practical reasons (e.g., administration time). The PGH-7 outperforms the PedsQL as a measure of overall health with regard to relative efficiency, which has several clinical implications. An often mentioned barrier of the use of PROMs in clinical practice is administration length. By using the PGH-7, less items are administered while obtaining similar reliable measurements. The PedsQL offers reliable measurements across a broader range of the scale compared to the PGH-7; however, this is mainly the case for participants that are more than three SDs from the mean (> 99th percentile). This could be considered a negligible benefit when taking into account the amount of additional items that need to be administered. In addition, it may not be necessary to have more information on patients that report a very poor overall health. If someone reports problems on all domains of overall health, additional questions may not provide more (useful) information about the health status of the respondent.

While this study demonstrated that the PGH-7 outperforms the PedsQL in terms of efficiency as a measure of overall health, it is important to acknowledge that both instruments contain different items and that the PedsQL reports subdomain scores, such as school functioning, which are not covered by the PGH-7. In clinical practice, the goal of the assessment has to match the chosen instrument based on the (item) content. The PGH-7 may not be a suitable replacement for domain-specific instruments as it has been developed to measure health at a higher, more global level.

It is peculiar that the general population in the Netherlands judge their global health to be slightly worse on average than the US population, whereas normative values on all scales of the PedsQL (except school functioning) are lower for the US [25] than for the Dutch general population [15]. This could be due to the phrasing of items as the items in the PGH-7 ask the participants to judge their own health, which may be influenced by cultural differences, such as self-criticism or high expectations of society.

The brevity of the PGH-7, while providing valid and reliable measurements, indicates that it could be adopted to assess pediatric overall health in research and clinical practice, as the administration time is only several minutes. As a generic PROM, the PGH-7 facilitates international comparisons and comparisons across patient groups and is now available for use in research and clinical practice in the Netherlands.

## Data Availability

Data will be made available upon reasonable request.

## Appendix A

**Table 2:** Sociodemographic characteristics of the study sample in comparison to the general population

	**8–12 years**				**13–18 years**		
**Pediatric study sample (*****N*** **= 492)**	**Sample (*****N***)	**Sample (%)**	**Dutch population*** **(%)**	**Adolescent study sample (*****N*** **= 606)**	**Sample** **(*****N*****)**	**Sample (%)**	**Dutch population* (%)**
**Age (years)**				**Age (years)**			
8	90	18.3	19.1	13	101	16.7	16.9
9	92	18.7	19.4	14	106	17.5	17.2
10	98	19.9	20.0	15	106	17.5	16.8
11	104	21.1	20.7	16	100	16.5	16.7
12	108	22.0	20.8	17	86	14.2	16.2
				18	107	17.7	16.3
**Gender**							
Male	241	51.0	51.1		316	52.1	51.1
Female	251	49.0	48.9		290	47.9	48.9
**Ethnicity**							
Dutch	386	78.5	76.0		510	84.2	76.3
Non-western immigrants	85	17.3	17.4		63	10.4	16.9
Western immigrants	21	4.3	6.6		33	5.4	6.8
				**Educational level**			
				Elementary^a^	6	1.0	0.6
				Lower vocational LBO/VMBO^a^	149	24.6	23.2
				Lower vocational MAVO^a^	189	31.2	30.1
				Secondary vocational MBO^b^	118	19.5	20.1
				General secondary education HAVO/VWO^c^	129	21.3	21.8
				HBO/WO Bachelor^c^	15	2.5	4.1
				WO Master or Doctorate^c^	0	0.0	0.2

*Based on the Gold Standard 2017 (Statistics Netherlands; www.cbs.nl/en-gb) population numbers

^a^ Low educational level

^b^Intermediate educational level

^c^High educational level

## Appendix B

**Table 3:** Assumption check for item response theory models

**Assumption**	**Analyses and criteria**		**Results**	**R package (version)**
**Unidimensionality** Unidimensionality refers to the ability of the measure to summarize the one, intended underlying trait (overall health) rather than two or more other underlying traits	Confirmative factor analysis (CFA) where all items load on a single, unidimensional factor	*CFI/TLI* > *0.95* *SRMR* < *0.10* *RMSEA* < *0.08*	0.97 0.07 0.19	lavaan (v0.6.3)
	Bi-factor analysis, where three random factors are added and general factor strength is calculated	*ω*_h_ > *0.80* *ECV* > *0.60* *G** > *0.30*	0.75 0.69 All G* > 0.30	psych (v2.1.9)
**Local independence** Local independence implies that besides both items of any pair measuring overall health, there is no other (statistical) relationship between the items	Item pairs should be independent after accounting for overall health; therefore, residual correlations of the CFA model are assessed per item pair	*Residual correlation between item pairs* ≤ *0.20*	All pairs ≤ 0.20	lavaan (v0.6.3)
**Monotonicity** Monotonicity implies that, on average, when someone has a higher overall health, a higher response is expected on each item in the scale than someone with a lower overall health would report	Mokken analysis to assess scalability of the total scale and each item	*Scalability coefficient H (scale)* > *0.50* *Scalability coefficient H*_*i*_* (item)* > *0.30*	*H* = 0.54 All item *H*_i_ > 0.30	mokken (v3.0.6)

*CFI*, comparative fit index; *TLI*, Tucker-Lewis Index; *RMSEA*, root mean square error of approximation; *SRMR*, standardized root mean square residual; *ω*_*h*_, hierarchical omega; *ECV*, explained common variance; *G**, general factor loading

## Abbreviations

CAT: Computerized adaptive test(ing)
CFA: Confirmatory factor analysis
CFI: Comparative fit index
DIF: Differential item functioning
ECV: Explained common variance
E-M: Expectation-maximization
HRQOL: Health-related quality of life
IRT: Item response theory
PedsQL: Pediatric Quality of Life Inventory
PGH: PROMIS Pediatric Global Health
PROM: Patient-reported outcome measure
PROMIS: Patient-Reported Outcomes Information System
RMSEA: Root mean square error of approximation
SD: Standard deviation
SE: Standard error
SRMR: Standardized root mean square residual
TLI: Tucker-Lewis index
US: United States

## Code availability

Custom code will not be made available.
